# Early Hospital Discharge Using Remote Monitoring for Patients Hospitalized for COVID-19, Regardless of Need for Home Oxygen Therapy: A Descriptive Study

**DOI:** 10.3390/jcm12155100

**Published:** 2023-08-03

**Authors:** Samy Talha, Sid Lamrous, Loic Kassegne, Nicolas Lefebvre, Abrar-Ahmad Zulfiqar, Pierre Tran Ba Loc, Marie Geny, Nicolas Meyer, Mohamed Hajjam, Emmanuel Andrès, Bernard Geny

**Affiliations:** 1 Physiology and Functional Exploration Service, University Hospital of Strasbourg, 67000 Strasbourg, France; bernard.geny@chru-strasbourg.fr; 2 Research Team 3072 “Mitochondria, Oxidative Stress and Muscle”, University of Strasbourg, 90032 Strasbourg, France; emmanuel.andres@chru-strasbourg.fr; 3 UTBM, CNRS, FEMTO-ST Institute, 90000 Belfort, France; sid.lamrous@utbm.fr; 4 Pneumology Department, University Hospital Strasbourg, 67000 Strasbourg, France; loic.kassegne@chru-strasbourg.fr; 5 Infectious Disease Department, University Hospital Strasbourg, 67000 Strasbourg, France; nicolas.lefebvre@chru-strasbourg.fr; 6 Internal Medicine Department, University Hospital Strasbourg, 67000 Strasbourg, France; abzulfiqar@gmail.com; 7 Public Health Department, University Hospital Strasbourg, 67000 Strasbourg, France; pierre.tranbaloc@chru-strasbourg.fr (P.T.B.L.); nicolas.meyer@chru-strasbourg.fr (N.M.); 8 Association for Assistance to Victims, Place Alfred de Musset, BP 3314, CEDEX, 27033 Evreux, France; mariegeny.mg@gmail.com; 9 Predimed Technology, 67300 Schiltigheim, France; mohamed.hajjam@predimed-technology.com

**Keywords:** COVID-19, sanitary crisis, home-telemonitoring, artificial intelligence, remote monitoring, early hospital discharge

## Abstract

Aim: Since beds are unavailable, we prospectively investigated whether early hospital discharge will be safe and useful in patients hospitalized for COVID-19, regardless of their need for home oxygen therapy. Population and Methods: Extending the initial inclusion criteria, 62 patients were included and 51 benefited from home telemonitoring, mainly assessing clinical parameters (blood pressure, heart rate, respiratory rate, dyspnea, temperature) and peripheral saturation (SpO_2_) at follow-up. Results: 47% of the patients were older than 65 years; 63% needed home oxygen therapy and/or presented with more than one comorbidity. At home, the mean time to dyspnea and tachypnea resolutions ranged from 21 to 24 days. The mean oxygen-weaning duration was 13.3 ± 10.4 days, and the mean SpO_2_ was 95.7 ± 1.6%. The nurses and/or doctors managed 1238 alerts. Two re-hospitalizations were required, related to transient chest pain or pulmonary embolism, but no death occurred. Patient satisfaction was good, and 743 potential days of hospitalization were saved for other patients. Conclusion: The remote monitoring of vital parameters and symptoms is safe, allowing for early hospital discharge in patients hospitalized for COVID-19, whether or not home oxygen therapy was required. Oxygen tapering outside the hospital allowed for a greater reduction in hospital stay. Randomized controlled trials are necessary to confirm this beneficial effect.

## 1. Introduction

The COVID-19 pandemic affected many patients worldwide, leading to millions of deaths and even more patients suffering greatly from long-COVID or post-COVID conditions. Globally, at the beginning of May 2023, there were 765,222,932 confirmed cases of COVID-19, including 6,921,614 deaths, reported to the WHO [1]. However, nearly 20 million persons might have died worldwide due to COVID-19, 5–15% of whom needed hospitalization. Further, approximately 10% of COVID-19 patients might demonstrate long-lasting symptoms, some of them until two years after the initial viral infection, which significantly limits both quality of life and ability to return to work [2,3,4,5]. This viral infection is not yet totally under control and rebounds, likely related to other variants, might result in care facilities becoming congested again, causing further harm to the population. Thus, even if Omicrons variants are generally less severe than the first variant, healthcare systems may be overwhelmed, particularly in countries with low population immunity [6]. Recently, in France, we still observed 422,226 contaminations by COVID-19 in March and April 2023 [1]. 

In October 2020, we observed, for the second time in France, an accelerated deterioration in COVID epidemic indicators, with many patients hospitalized for COVID-19 every day. The national 7-day incidence rate was above 430 per 100,000 inhabitants (Public Health France). To limit viral transmission in all territories, and thus reduce the impact on the hospital capacity as quickly as possible, the French government decided to take drastic measures, including a population lock-down. Nevertheless, it was necessary to adapt our healthcare system to avoid overflows in critical care hospitalization capacity and preserve conventional hospitalization capacity. Indeed, this is a major issue in the case of severe forms requiring hospitalization [7]. 

Spare hospital bed vacancies might be obtained by strengthening the link between conventional hospitalization and community medicine, aiming to allow for selected patients to be discharged from hospital earlier. Retrospectives studies have supported the potential usefulness of telemonitoring and telemedicine in COVID-19 patients, allowing for an early return home [8,9,10].

To this end, a national strategy for facilitated and adapted post-hospital care was implemented within the framework of a coordinated care pathway. We regionally developed TELECOVID-RAD, which is an innovative telemonitoring system set up to favor an early return home for patients hospitalized in the Strasbourg University Hospital for SARS-CoV-2, whether or not oxygen therapy was required. This solution, part of the healthcare pathway, re-enforced the city–hospital link to facilitate the hospitalization/discharge process. This offered healthcare providers the opportunity to closely monitor symptoms and vital parameters of COVID-19 patients in their own homes.

The aim of this prospective and descriptive study was to evaluate a newly created telemonitoring coordinated-care pathway in the setting of the COVID-19 pandemic’s resurgence. We investigated whether patients requiring home oxygen therapy might also safely benefit from an early hospital discharge, as presumed in patients not requiring oxygen [11], and whether this can reduce the duration of hospitalization without readmission, facilitating a faster turn-over of beds and thus providing the possibility of other patients being hospitalized.

## 2. Population and Methods

### 2.1. Population and Study Design

#### 2.1.1. Patients’ Inclusion and Exclusion Criteria

Patients presenting with COVID-19 RT-PCR-confirmed infection and oxygen saturation below 93% on ambient air, whether or not this was associated with major dyspnea, who were hospitalized in the University Strasbourg Hospital, France, from November 2020 to August 2021, were proposed for participation in this prospective study. All patients gave their informed consent, and the study was approved by the ethics committee of the University of Strasbourg (CE-2022-61, 2 October 2022).

For all patients, inclusion criteria related either to their environment or to their clinical characteristics. Thus, a fixed and sanitary home less than 30 min from the referral health care institution with a nearby emergency structure and the possibility to isolate in a single room, together with the presence of a third party 24 h a day/7 days a week with reliable telephone access, were mandatory. For patients still requiring oxygen at home, specific inclusion criteria were autonomy (Katz ADL > 3/6) and oxygen requirement < 4 L/min (nasal cannula or mask) to maintain pulsed oxygen saturation (SpO_2_) > 92% at rest. 

The French High Heath authority (HAS) initially stated that patients needing home oxygen therapy and presenting with one major or more than one minor exclusion criteria should not be included in home telemonitoring. The major exclusion criteria were refusal of the patient, his entourage or his attending physician, absence of inclusion criteria, destabilized chronic pathologies, cancer under chemotherapy, congenital or acquired immune-depression, solid organ malignancy, hematopoietic stem cell transplantation related to a hematological malignancy under treatment, decompensated cirrhosis, neurological or neurovascular disease that may impair respiratory function, morbid obesity (body mass index-BMI ≥ 40 kg/m^2^), suspicion of pulmonary embolism or pulmonary embolism not excluded (positive clinical and D-dimer arguments), or confirmed pregnancy regardless of term. 

Minor exclusion criteria were numerous: >70 years old, severe cardiovascular pathologies (arterial hypertension with polytherapy, history of stroke or coronary artery disease, heart surgery, heart failure), balanced diabetes, chronic respiratory pathology, cancer controlled under treatment including radiotherapy < 6 months, no decompensated cirrhosis, moderate to severe obesity (body mass index (BMI) ≥ 30 and <40 kg/m^2^). These criteria were designed to encourage patients with the highest risk of severe forms to stay in hospital rather than return home early. Nevertheless, as part of a doctor–patient shared decision, home oxygen therapy was considered regardless of age (HAS 9 November 2020). Further, in view of the numerous comorbidities present in severe forms of COVID-19, we also proposed home telemonitoring to stabilized patients with more than one minor exclusion criteria, considering their specific health status and environmental context.

The medical team decided on early discharge based on significant clinical improvements in patients, and the stability of this amelioration over time.

#### 2.1.2. Telemonitoring Establishment

At least 24 h before the patient’s hospital discharge, after obtaining informed consent, the attending hospital physician notified the family doctor and the regional care-coordination platform (regional support platform (PRAG)). The platform was then responsible for coordinating care at home with the patient’s healthcare professionals (general practitioner (GP), nurses, physiotherapists, and pharmacists if oxygen therapy was required), as well as the provider of the remote monitoring equipment.

**Figure 1:**
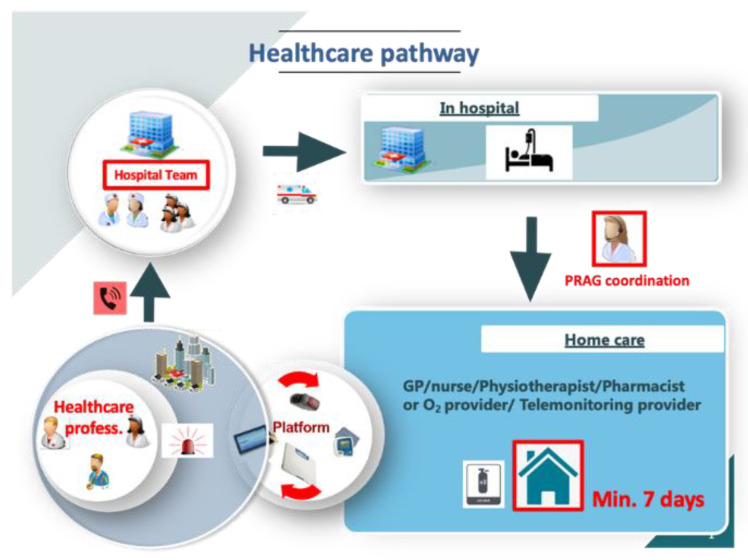
Telemonitoring organization. GP: general practitioner; Healthcare profess.: healthcare professional; PRAG: healthcare regional support platform.

A technician from the remote monitoring solution came to the patient’s home on the day of discharge from hospital to install remote monitoring devices (connected devices such as an oximeter, blood pressure monitor and tablet, approved for medical use in Europe). The patient, a caregiver, and the nurse were trained in their use and if there was no WIFI at home, a substitute solution using a 4G key was provided (Figure 1).

Home care of patients started in the immediate aftermath of hospitalization for 7 days (extendable according to the advice of the attending physician). The nurse received alerts at the patient’s bedside via the tablet present in the home, or remotely on his/her smartphone when a relative took measurements. The nurse called the attending physician (GP) in case of an alert.

The telemonitoring care pathway ended when patients no longer needed home monitoring because symptoms sufficiently improved or oxygen weaned, or if patients needed admission to the hospital.

#### 2.1.3. Parameters Determined and Alerts Settings

Patients on oxygen therapy measured the following parameters three times a day: respiratory rate, heart rate, temperature, systolic and diastolic blood pressures, and SpO_2_. Patients without oxygen therapy performed these measurements once a day. Connected sensors allowed for the telemonitoring of blood pressure and oxygen saturation, and questionnaires on symptoms (dyspnea using NYHA scale, presence or absence of chest pain, shivers) were filled in daily on the tablet by the patient or his caregiver. 

**Figure 2:**
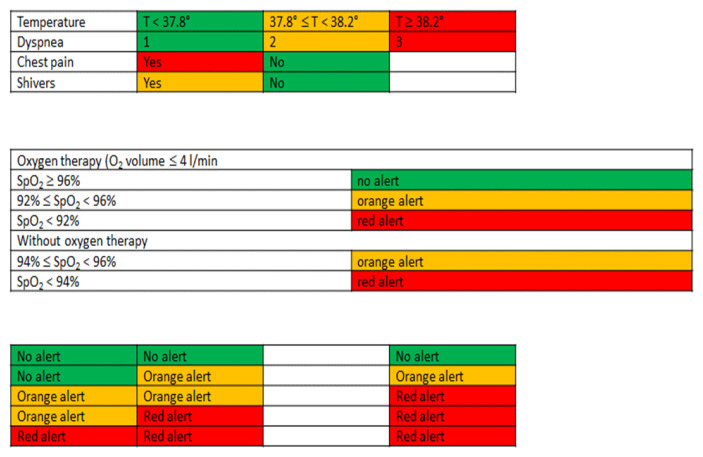
Clinical signs and SpO_2_ rating for the generation of alerts. One green alert combined with one orange alert generates an orange global alert; two orange alerts generate a red global alert; one red alert combined with one orange alert generates a red global alert; two red alerts generate a red global alert.

The artificial intelligence platform considered all the parameters to allow for an analysis and trigger eventual alerts. Alerts were color-coded as green (low risk), orange (intermediate risk) or red (high risk), and then automatically sent to the smartphone of the patient’s doctor and nurse each day (Figure 2).

#### 2.1.4. Patient’s Satisfaction Evaluation

To further determine the usefulness of the remote monitoring of COVID-19 patients, we evaluated patient satisfaction using a questionnaire with three simple key questions. Patients quantified whether they found the device simple to use, whether they felt safe, and whether the care coordination was satisfying on a scale ranging from 1 to 5, with 1 being the lowest and 5 being the highest satisfaction. Data are presented as the mean value.

### 2.2. Statistical Analysis

Qualitative data were described with frequency (%) and quantitative data, with the mean (SD) or median [Q1–Q3]. Kaplan–Meier estimators were used to evaluate time to resolution of dyspnea and tachypnea. Categorical data were compared with the chi^2^ test with no continuity correction. Quantitative data were compared with *t*-test. The level of significance was set at 0.05. All computations were carried out with R 4.2.2.

## 3. Results

### 3.1. Flow Chart of the Study

**Figure 3:**
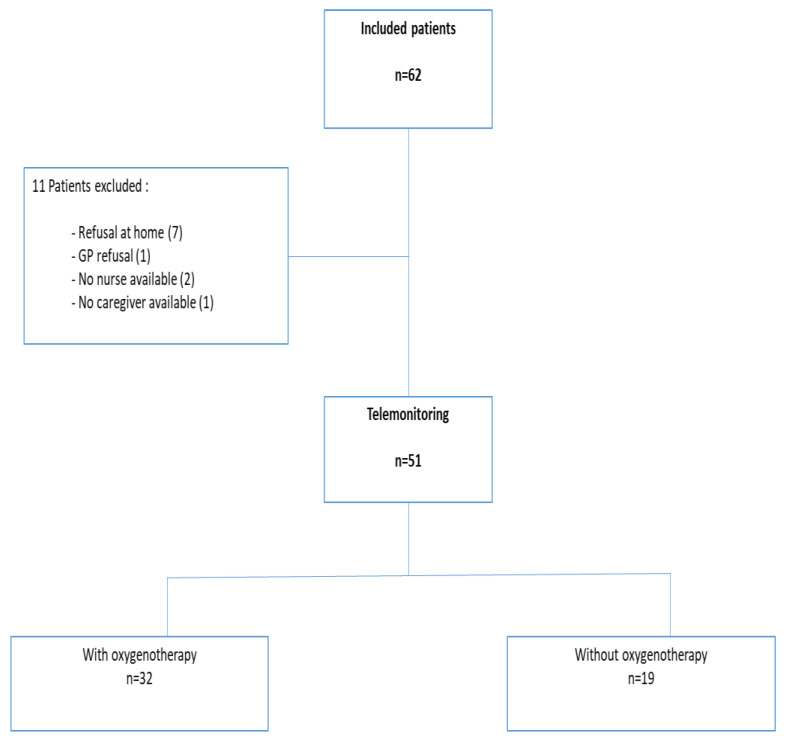
Flow chart of the study.

Of the 1834 patients hospitalized in our institution for COVID-19 from November 2020 to August 2021, 62 were included by the regional support platform (PRAG) and 51 were home telemonitored after hospital discharge with My Predi^®^ (Predimed Technology, 667300 Schiltigheim, France) solution. Eleven patients discontinued treatment, mainly due to refusal to continue upon arriving home, or because caregivers were not sufficiently available. Among these 51 patients, 32 (63%) were still receiving nasal oxygen at hospital discharge (Figure 3).

### 3.2. Patients’ Clinical Characteristics, Main Risk Factors, Comorbidities and Symtoms

The mean age of the patients was 65 years, and 2 (4%), 25 (49%) and 24 (47%) of them were below 45, between 45 and 65 and older than 65 years old, respectively. There were slightly more men than women (55% versus 45%), and mean BMI was 28.6 kg/m^2^, ranging from 16.9 to 41.7. Fourteen (27.5%) patients were obese with a BMI > 30 kg/m^2^. 

The main risk factors and co-morbidities were hypertension (55%), diabetes mellitus (29%), and obesity (27%). Ninety-eight percent of the patients presented with comorbidities, and 63% of them presented with more than one minor comorbidity (Table 1). Charlson’s indice of comorbidities was 4.2 (±2.7), despite the absence of active neoplasia in our population (exclusion criteria). Thirty-three percent of the patients were transiently hospitalized in a critical care unit upon their arrival at hospital.

### 3.3. Patients’ Evolution at Home

#### 3.3.1. Need for Re-Hospitalization

In our cohort, two patients (4%) were re-admitted to the hospital on day 15 after discharge. One, related to chest pain occurrence, resumed spontaneously, and no cause was found. This patient was discharged from hospital on the same day as readmission. The second patient presented with a pulmonary embolism detected by the platform during home telemonitoring and was re-hospitalized with a favorable clinical evolution. No other significant deleterious situations occurred and no death occurred within 30 days of hospital discharge.

#### 3.3.2. Evolution of Clinical Parameters during Home Telemonitoring

As presented in Table 2, temperature and systemic blood pressure, collected in 51 patients, were in the normal range and stable during home telemonitoring. Very few chests pain or shivering episodes occurred.

The platform raised a total of 1238 alerts, including 722 (58%) red and 505 (41%) orange alerts. No at-home life-threatening emergencies occurred.

**Figure 4:**
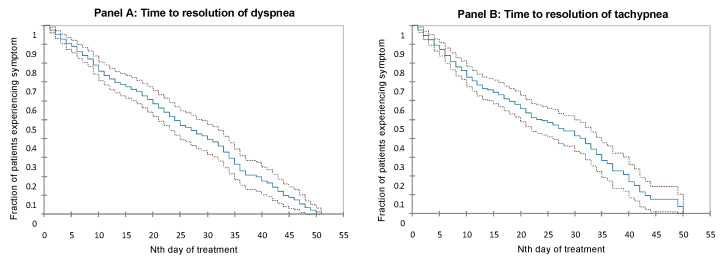
Kaplan–Meier curves showing the time-course of dyspnea (**Panel A**) and tachypnea (**Panel B**) resolutions in patients requiring home oxygen therapy. Blue lines show the fraction of patients still presenting dyspnea (A) and/or tachypnea (B) over time and the black lines indicates the 95% confidence interval of time to symptom resolution.

The longest symptoms were dyspnea and tachypnea. The mean time to dyspnea resolution was 21 ± 1 days and 24 ± 1 days in the entire population and in the patients discharged with oxygen, respectively (Figure 4A). The mean time to tachypnea resolution was 23 ± 1 and 24 ± 1 days in the entire population and in the patients discharged with oxygen, respectively (Figure 4B). Among the 19 patients discharged without oxygen therapy, no patients needed new oxygen during the home telemonitoring period. 

#### 3.3.3. Oxygen Therapy Evolution during Home Telemonitoring

The data concerning oxygen therapy are presented in Table 3.

Mean oxygen output at hospital discharge and during home telemonitoring was below 2 L/min. Mean SpO_2_ during home telemonitoring was 95.7 ± 1.6%.

For all patients weaned from oxygen at home, mean and median oxygen weaning durations were 13.3 ± 10.4 days and 9.5 (1–41) respectively. The cumulative total of days required for oxygen weaning (duration +1 day) was 430 days.

#### 3.3.4. Patients Satisfaction Evaluation

Thirty-six patients filled out the questionnaire and their assessment of the device’s simplicity to use, their feeling of safety and their satisfaction with the care coordination team was 3.7, 3.6, and 3.8/5. 

#### 3.3.5. Number of Saved Hospital Days Due to Telemonitoring

The mean duration of telemonitoring was 9.2 ± 3.5 and 17.8 ± 13.7 in patients without and with home oxygen therapy, respectively. This corresponds to 743 hospital days being saved due to telemonitoring; with most of these (568 days) being related to patients needing oxygen therapy.

## 4. Discussion

The main results of this prospective study demonstrate the safety of an early return home of patients hospitalized for COVID-19. Although patients older than 65 years, those presenting with comorbidities, and/or those requiring oxygen therapy were included, no deaths and only two readmissions occurred within the 30 days following hospital discharge. Further, 743 potential days of hospitalization were saved for other patients, with the majority being related to the early return home of patients still under oxygen. 

### 4.1. Feasibility of Early Return Home in Patients Hospitalized for COVID-19

At the time of the study, doubts were raised concerning the feasibility of an early return home in patients hospitalized for COVID-19. Fears of aggravation were particularly strong when oxygen therapy was mandatory. Accordingly, although the need for beds was high in view of the exploding pandemic, restrictive inclusion criteria were proposed to select patients for whom early hospital discharge might be proposed. The aim was to limit the risk of re-admission to hospital, potentially due to an emergency. Thus, initially, the inclusion criteria set by the high health authority (HAS) were quite strict, and a single major criterion or more than one minor criteria excluded patients from this early return home protocol. 

These HAS recommendations define good practices, whose purpose is to guide healthcare professionals in designing and implementing the most appropriate preventive, diagnostic or therapeutic care strategies on the basis of proven medical knowledge at the date of their publication [12]. Accordingly, these recommendations evolve with improvements in knowledge.

Although comorbidities likely aggravate COVID-19 patiens’ prognosis [13], in view of our population characteristics, we decided to include patients who presented with more than one minor exclusion criteria in cases of clinical stabilization. Indeed, strictly following these exclusion criteria or simply waiting for their official implementation would have excluded many patients from the potential benefits of an early return home. This shared decision greatly improved the clinical feasibility of the study, without any deleterious effects.

Home localization and the need for a rigorous follow-up for patients at home may also reduce the feasibility of such a protocol. Indeed, not all patients live near a hospital or emergency structure, and these patients could benefit from the daily attentions of a relative or available caregivers.

Nevertheless, despite these potential limitations, previous studies reported that an early return home could be proposed for patients suffering from severe forms of COVID-19. Indeed, an overflow of critical care hospitalization capacity occurred in many countries [7]. Retrospective analyses were, therefore, conducted to investigate the feasibility of telemedicine. TELEA in Galicia, a proactive at-home monitoring of patients considered to be high-risk, was associated with lower rates of hospitalization, shorter hospital stays, a lower mortality rate in the first hospitalization, and no at-home life-threatening emergencies [10]. In a primary healthcare center in Barcelona within the context of the first wave of the pandemic—when no rapid diagnostic tests were available—telephone follow-up by healthcare professionals was effective in detecting progression to severe COVID-19 and pneumonia, diagnosing more than 80% of the cases of pneumonia in non-severe COVID-19 patients [14].

In March 2020 in Wisconsin, Annis et al. reported, in 2255 patients, the quick implementation of a remote patient-monitoring program as an effective approach for managing COVID-19 symptoms at home [15]. In France, Covidom was a large telemonitoring solution that was rapidly deployed in the greater Paris area in 70,914 patients during the first wave of the pandemic to monitor patients with COVID-19 at home [16]. This early large-scale solution combined a free web application allowing for patients to fill out short daily questionnaires on their health status with a regional control center that monitored and managed the alerts triggered by questionnaire responses. This solution efficiently alleviated the burden on health care systems’ capacities, and cumulatively mobilized more than 2000 volunteers, mostly specialists with decreased activity due to the first lockdown. In contrast, during this wave of the pandemic in France, when hospital activity dramatically increased due due to the preservation of surgery activity while COVID-19 cases progressed, our home system did not require hospital staff and allowed for patients to be efficiently cared for and monitored remotely by their regular GP and nurses at home.

### 4.2. Safety of Early Return Home in Patients Hospitalized for COVID-19

In the early stages of COVID-19, the safety of this measure has been questioned in view of the gravity of the COVID-19 disease and patients’ potential aggravation, particularly when oxygen therapy is needed. In our study, importantly, safety was good in patients, regardless of the requirement for oxygen therapy. Thus, the only two re-admissions at hospital were not directly due to virus-related aggravations. Rather, telemonitoring likely allowed for an earlier diagnosis of pulmonary embolism, detected by the platform during home telemonitoring. Our data are in line with previous studies supporting the safety of an early return home in patients suffering from COVID-19 [10,14,15,16].

Of particular interest, the need for oxygen therapy, and the increasing length of hospitalization until oxygen weaning, suggests that patients are more vulnerable. However, our results are in line with Covidom in the subgroup of patients requiring nasal oxygen therapy [17] and with Banerjee et al.’s study in the USA, which showed that patients discharged with home oxygen had low rates of mortality and re-hospitalization within 30 days of discharge, preserving safe access to acute care during the COVID-19 pandemic [18]. In the Netherlands, Grutters et al. showed, using retrospective data, that early discharge was possible in severe COVID-19 patients if home telemonitoring included the use of pulse oximetry [19]. Further, van Herwerden et al. showed in 49 patients, that, although 6 patients required readmission, home telemonitoring and oxygen administration can be safely applied in COVID-19 patients [20]. Very interestingly, another study comparing home telemonitoring and oxygen therapy started directly after emergency department (ED) vs. hospital admission, concluded that starting home telemonitoring and oxygen therapy directly after ED assessment was safe [21], with a total home telemonitoring duration of 14 days in both groups.

Thus, early returns home in patients hospitalized for COVID-19, whether or not oxygen is required, appears to be quite safe.

### 4.3. Usefulness of Early Return Home in Patients Hospitalized for COVID-19

Besides allowing for more personalized care to be delivered to patients, early returns home aimed to avoid hospital overflow, and thus to allow for other patients, whether or not they were suffering from COVID-19, to be hospitalized as needed. Indeed, numerous patients may not have always benefited from an adapted and timely therapy because of the unavailability of the hospital structure during the COVID-19 pandemic. This pitfall will probably also apply in future pandemics, whatever their origin.

Importantly, in our study we observed 17.8 days and 568 days, respectively, of median duration and cumulative days of home telemonitoring in our oxygen sub-group. This is similar to van Herwerden MC’s data, showing a potential reduction in hospitalization of 616 days [11]. Similarly, Grutters LA et al. observed that the greatest reduction in hospitalization duration was seen in patients needing home oxygen therapy [10]. Taken together, our clinical protocol appeared efficient; the 743 cumulative days of home monitoring (of which 430 days were needed for oxygen weaning for 32 patients), saved a minimum of 49 hospital beds (based on a COVID-19-related mean hospitalization length of 15.2 days in our hospital). This is consistent with the results of Hanninen (in press) and Suárez-Gil et al., who found shorter hospital stays and lower readmission rates in COVID-19 patients followed at home by telemonitoring [22].

A usefulness assessment including questionnaires on patient’s satisfaction and assessments of mental stress and anxiety as an outcome measure would have been interesting. Indeed, patients might experience higher anxiety when managing significant equipment at home without the presence of a doctor, compared to being in the hospital with readily available healthcare professionals. Although not mental stress and anxiety were not specifically investigated, we observed a fair level of satisfaction in our patients. Indeed, a majority found the device simple to use and the organization to be efficient, and accordingly felt safe with at-home monitoring. Our results are consistent with previous data showing high satisfaction levels in telemonitored COVID-19 patients [20]. 

### 4.4. Study Limitations and Perspectives

COVID-19 patients’ duration of hospitalization was not compared to that of a control group without telemonitoring. A case-control design with a comparison group matching the characteristics of patients still admitted in the hospital would have strengthened the study. While patients on oxygen might fare better at home compared to those discharged without oxygen, this cannot be guaranteed by our design, supporting the need for complementary studies.

Nevertheless, when the length of stay in our hospital of 1756 patients discharged without telemonitoring during the same period was noted, we inferred an important reduction in days spent in a hospital bed (49 days). With the current decline in the number of hospital healthcare professionals, this study shows that this home telemonitoring system is suitable and applicable in the event of hospital tensions linked to strong epidemic periods, such as seasonal flu, which would necessitate increased hospitalization facilities for patients requiring oxygen therapy. 

A prospective study including a dedicated nurse coordination unit receiving alerts directly from the homecare remote monitoring system, who could contact patients and their healthcare professionals in return, could further improve the efficiency and safety of homecare. This future study would also allow for a comprehensive implementation analysis, including interviews with doctors, teleproviders, and caregivers, in order to improve the design of this useful help in patient care.

## 5. Conclusions

This prospective study demonstrates that the remote monitoring of vital parameters and symptoms allows for early hospital discharge in patients hospitalized for COVID-19, whether or not home oxygen therapy is required. Extending these criteria to include patients presenting with more than one comorbidity did not reduce safety.

Interestingly, patients requiring home oxygen therapy likely benefitted more from TELECOVID-RAD, since it allowed for oxygen tapering to occur outside the hospital, and thus a greater reduction in hospital stay compared to patients who did not require oxygen therapy.

Nevertheless, even if our data support the idea that home monitoring reduces the hospital stay of patients with COVID-19, randomized controlled trials are necessary to confirm these beneficial effect [23] and demonstrate that more beds are thus available for other patients. Indeed, this important parameter during sanitary crises also depends on the medical and paramedical team’s capacity to take care of more patients.

## Figures and Tables

**Table 1:** COVID-19 patients’ clinical characteristics, risk factors, comorbidities and symptoms.

		Total Population (*n* = 51)	Patients with Oxygen (*n* = 32)	Patients without Oxygen (*n* = 19)	*p*-Value
**Clinical** **characteristics**	Age, years Mean (±SD)	64.9 (±14)	65.5 (±15)	63.9 (±12)	0.677
	Male, *n* (%)	28 (55%)	16 (50%)	12 (63%)	0.361
	Female, *n* (%)	23 (45%)	16 (50%)	7 (37%)	
	BMI, kg/m^2^ Mean (±SD)	28.6 (±5)	27.2 (±5)	30.9 (±5)	0.009
**Main risk factors and comorbidities**	Hypertension, *n* (%)	28 (55%)	17 (53%)	11 (58%)	0.741
	Diabetes, *n* (%)	15 (29%)	7 (22%)	8 (42%)	0.125
	Obesity, *n* (%)	14 (27%)	7 (22%)	7 (58%)	0.247
	Coronaropathy, *n* (%)	9 (18%)	6 (19%)	3 (16%)	0.789 *
	Active smoking, *n* (%)	8 (16%)	5 (16%)	3 (16%)	0.988 *
	Asthma, *n* (%)	4 (8%)	1 (3%)	3 (16%)	0.190 *
	Chronic obstructive pulmonary disease, *n* (%)	1 (2%)	0 (0%)	1 (5%)	0.104 *
**Symptoms**	Dyspnea, *n* (%)	43 (84)	28 (88)	15 (79)	0.417
	Weakness, *n* (%)	40 (78)	25 (78)	15 (79)	0.945
	Cough *n* (%)	38 (75)	27 (84)	11 (58)	0.036
	Digestive troubles, *n* (%)	20 (39)	14 (44)	6 (32)	0.389
	Anorexia, *n* (%)	15 (29)	10 (31)	5 (26)	0.708
	Myalgias, *n* (%)	12 (24)	8 (25)	4 (21)	0.748
	Headache, *n* (%)	9 (18)	7 (22)	2 (11)	0.304
	Anosmia/dysgeusia, *n* (%)	6 (12)	3 (9)	3 (16)	0.492
	Thoracic pain, *n* (%)	4 (8)	2 (6)	2 (11)	0.583

All tests: chi^2^ test with no continuity correction. *: Fisher exact test gave the same conclusion as to significance.

**Table 2:** Telemonitored clinical and functional signs and alerts.

Temperature and Systemic Blood Pressure	First Day	Last Day
Temperatures (°C), mean (±SD)	36.6 (±0.4)	36.6 (±0.5)
BP (mmHg), mean (±SD) Systolic	122 (±15)	125.2 (±17)
Diastolic	76.1 (±13)	79.8 (±14)
Mean	83.5 (±15)	80.9 (±16)
**Functional Signs (*n* = 41)**		
Chest pain: no/increasing/decreasing, *n* (%)	36 (88)/2 (5)/2 (5)	
Shivers: no/yes, *n* (%)	40 (98)/1 (2)	
**Alerts, *n* (%)**		
Cumulative: Total/Red/Orange	1238 (100)/722 (58)/505 (41)	
Mean per patient: Total/Red/Orange	24.1/14.2/9.9	

Cpm: cycle per minute; °C: Celsius, mmHg: millimeters of mercury, BP: blood pressure.

**Table 3:** Oxygen therapy evolution during home telemonitoring.

	Patients with Oxygen *n* = 32
Oxygen output (L/min) at hospital discharge mean (±SD)	1.9 (±0.7)
Mean oxygen output (L/min) during home telemonitoring mean (±SD)	1.6 (±0.7)
Oxygen weaning achievement *n* (%)	32 (100%)
Oxygen weaning duration, in days mean (±SD)	13.3 (±10.4)
Oxygen weaning cumulative days	430

## Data Availability

Data are available by asking to the corresponding author.
